# The Uses of Herbaria in Botanical Research. A Review Based on Evidence From Argentina

**DOI:** 10.3389/fpls.2019.01363

**Published:** 2019-11-07

**Authors:** Alicia López, Agostina B. Sassone

**Affiliations:** ^1^Facultad de Ciencias Agrarias, Universidad Nacional de Mar del Plata, CONICET, Balcarce, Argentina; ^2^Instituto de Botánica Darwinion, CONICET-ANCFEN, Buenos Aires, Argentina

**Keywords:** Argentina, data mining, natural sciences, scientific institution, social sciences

## Abstract

Botanists, a section of the broad universe of researchers in Biology, are intensive users of herbaria. Presumably, all botanists use herbaria, with greater or lesser frequency and intensity, in the development of their research. In this article, we will try to prove this statement. For this purpose, an institutional history of Botany and herbaria in Argentina is presented. This study will also show that there are other fields of knowledge in which the herbarium has a role as an input, or data source, for research (e.g. agronomy, ethnobotany, medicine). On the other hand, it will be demonstrated that, in addition to the uses of the herbarium in basic science, this institution has a crucial role in the knowledge and preservation of biodiversity, and in the improvement of species for commercial use.

## Introduction

### Overview: The Historical Development of Herbaria in Argentina

Across the globe, there are 3,400 herbaria store botanical collections which are an invaluable record of the world’s biodiversity (Krishtalka et al., 2016; Thiers, 2019). Herbaria have unique advantages over other botanical repositories such as germplasm banks or botanical gardens in producing good quality data for native flora conservation, among other uses (Funk, 2018).

Plant collections started early in the Argentine territory, long before the organization of the Republic. Since the 1700s, European naturalists came in expeditions and collected the first Argentine specimens, which were then sent abroad in order to identify them and are still stored at foreign herbaria (e.g. for *Oxalis* L., endemic species from Argentina, the type specimens are stored in B, E, K, P and, S and for Amaryllidaceae, in B, BM, G, GOET, K, LY, among others; for acronyms see **Table 1**).

**Table 1:** List of acronyms of the mentioned herbaria, following Thiers (2019), continuously updated.

ANGU	Instituto Nacional de Tecnología Agropecuaria. Anguil. La Pampa. Argentina.
B	Botanischer Garten und Botanisches Museum Berlin-Dahlem, Zentraleinrichtung der Freien Universität Berlin. Berlin. Germany.
BA	Museo Argentino de Ciencias Naturales “Bernardino Rivadavia”. Buenos Aires. Argentina.
BAA	Universidad de Buenos Aires. Buenos Aires. Argentina.
BAB	Instituto Nacional de Tecnología Agropecuaria. Castelar. Buenos Aires. Argentina.
BAF	Universidad de Buenos Aires. Buenos Aires. Argentina.
BAL	Universidad Nacional de Mar del Plata - Instituto Nacional de Tecnología Agropecuaria. Balcarce. Buenos Aires. Argentina.
BM	The Natural History Museum. London. England. U.K.
CORD	Museo Botánico. Córdoba. Argentina.
CTES	Instituto de Botánica del Nordeste. Corrientes. Argentina.
E	Royal Botanic Garden Edinburgh. Edinburgh. Scotland. U.K.
G	Conservatoire et Jardin botaniques de la Ville de Genève. Genève. Switzerland.
GOET	Universität Göttingen. Göttingen. Germany.
K	Royal Botanic Gardens. Kew. England. U.K.
LIL	Fundación Miguel Lillo. Tucumán. Argentina.
LP	Museo de La Plata. La Plata. Buenos Aires. Argentina.
LY	Université Claude Bernard. Lyon. France.
MO	Missouri Botanical Garden. Saint Louis. Missouri. U.S.A.
NY	The New York Botanical Garden. Bronx. New York. U.S.A.
P	Muséum National d’Histoire Naturelle. Paris. France.
S	Swedish Museum of Natural History. Stockholm. Sweden.
SI	Instituto de Botánica Darwinion. San Isidro. Buenos Aires. Argentina.

Considering only the herbaria included in official institutions, the first one was the BA Herbarium, dated in 1854 as part of the Argentine Museum of Natural Sciences, followed by CORD in 1870 and LP in 1887. Nowadays, there are 47 active herbaria, being LIL (720,000 entries), SI (700,000), CTES (530,000), LP and CORD (both with 500,000) the most important ones.

Nowadays, the active collectors store their materials in local herbaria (such as BAB, CTES, SI) although the international collaboration stimulates to send duplicates abroad (e.g. B, MO, NY). To date, Argentina holds ca. 5 million herbarium specimens stored at 47 active herbaria distributed in 16 out of 23 provinces (for a detailed information about Argentinian Herbaria please refer to Zardini, 1980).

### Herbaria and the Development of Botany

The history of Botany in Argentina begins with the arrival of the Jesuits during the times of the Spanish conquest, and follows with the organization of the nation-state when the European naturalists hired by the first rulers could carry out their studies in the country and contribute in organizing local institutions (de Asúa., 2012). It is interesting to note that, with the formation of modern nation-states in Latin America, the lands of the indigenous people became part of the national territory of the newly constituted countries. The delimitation of their borders resulted in some indigenous people that lived outside of the major cities being distributed into two or more countries. This is the case of the Aymara, Mapuche, Guaraní, and Wichi people, among others. Consequently, the knowledge the indigenous people had about the flora was at first underestimated by academia, at least in the available records. Only in the latter decades has local knowledge been recovered (e.g. Pirondo et al., 2018; Suárez 2019). Nevertheless, when referring to the history of herbaria and the consolidation of scientific institutions, it is a necessary to go back to the moment of the establishment of Argentina as a nation-state.

During the eighteenth century, the natural sciences in the Viceroyalty of the Río de la Plata region (currently Argentina, Bolivia, Paraguay, Uruguay, southern Brazil, northern Chile, and southeast Perú) first progressed thanks to the Jesuit naturalists. Among them, Father Gaspar Xuárez is considered the first Argentine native botanist. In 1767, the Jesuit Order was expelled from America and Father Xuárez settled in Italy to continue his botanical studies, influenced by European botanists such as Cavanilles, de Jussieu, Ruiz, and Pavón. His most important botanical works are the three fascicles of Osservazioni Fitologiche, published in collaboration with Gilli in Rome in 1789, 1790 and 1792. The most notable feature of his study was that the binomial nomenclature was properly applied (Parodi, 1961; Sayago, 1972).

In 1812, the creation of the Museum of Natural History of Buenos Aires (current Argentine Museum of Natural Sciences) carried out by Rivadavia was the constitutive act of the Natural Sciences in the region. In 1816, Rivadavia traveled to Europe for diplomatic reasons and he was also commissioned to hire “illustrious men” to come and spread science in this country. As a result, Humboldt’s famous partner Bonpland, as well as Tweedie, Lorentz, Hieronymus, and Niederlein, were hired (De Asúa., 2012). Later on, foreign botanists arrived in the country, dedicated themselves to the Argentine flora, and strongly influenced the development of the discipline throughout the participation in scientific societies and as part of the numerous botanical institutes that were emerging (the most notable cases are summarized in **Table 2**). Since then, Botany emerged and settled down as a distinct discipline, strongly influenced by European naturalists.

**Table 2:** Notable European naturalist that work in Argentine Flora.

John Tweedie	1775, Lanarkshire, Scotland	1862, Buenos Aires, Argentina
Karl Hermann Konrad Burmeister	1807, Stralsund, Germany	1892, Buenos Aires, Argentina
Prof. Paul Günther Lorentz	1835, Kahla, Germany	1881, Concepción del Uruguay, Argentina
Georg Hans Emmo Hieronymus	1845, Silesia, Germany	1921, Berlin, Germany
Fritz Kurtz	1854, Berlin, Germany	1921, Cordoba, Argentina
Gustav Niederlein	1858, Berlín, Alemania	1924, Santiago del Estero, Argentina
Carlo Luigi Spegazzini	1858, Bairo, Italy	1926, La Plata, Argentina

Institutional change shapes the way societies evolve over time, and thus it is the key to understanding historical change (North, 1990). Until the first half of the 20th century, scientific activities in Argentina were concentrated in research centers such as universities, museums, and scientific academies, which had been created—or recreated—during this period. From these institutions, there emerged the first recognizable local scientists, who made it possible to reach a degree of maturity and international recognition. Two symbols of this recognition are the Nobel Prizes awarded to local scientists Bernardo Houssay, Luis Federico Leloir and César Milstein, and the current position of CONICET in international rankings (Miguel et al., 2007; e.g. https://www.scimagoir.com/rankings.php?sector=Government).

By the mid-1950s, the scientific activities were organized and, in 1958, the creation of the National Council of Scientific and Technological Research (hereon CONICET, as the acronym in Spanish) was a milestone in Argentine science. Thereby, the field of Botany as it is known today was settled, as professional investigators started dedicating full time to research affiliated to public institutions (ca. 70% of the scientific research is currently performed in public institutions such as university departments and governmental institutions). As the institutions and research activities were organized, so were the herbaria and their uses. For instance, a classic botanist would devote her or his entire life to revising a specific plant group through collecting, identifying and illustrating the material, and elaborating then the identification keys to final publish the manuscript. On the contrary, a modern taxonomist would be more of a generalist and delegate some of the activities. Consequently, classical herbaria would expand their functions to house new collections such as dried leaves in silica gel, lyophilized DNA, or digital images. In the last two decades, these changes have been reinforced as a result of the implementation of public policies promoting strategic lines of scientific research.

In their ordinary functioning as institutions, Herbaria have a flow of people and materials—specimens, illustrations, and more recently dry material in silica gel and DNA extractions—through loans, in which researchers affiliated to an institution request material from others to carry out their research. In recent years, the possibility of visiting virtual herbaria generated another type of relationship between researchers and objects. Nevertheless, the exchange of materials and people is irreplaceable, since it produces a movement of ideas, strengthens professional and personal relationships and stimulates new lines of scientific development: new questions, new approaches, new comparative studies.

Herbaria have their own rules: exchanges are drawn only between recognized herbaria and the researcher must have an affiliation to an institution in order to be allowed to ask for a loan. When visiting an herbarium, it is necessary to have an authorization to access collections and, in some places, there are specific protocols to look at the type specimens. There are also indications about how to annotate the specimens. Two researchers apart in time and space can interact on the same specimen. All these rules—explicit or not—model the botanical research activity.

## Data Mining in Local and Global Databases

With the intention of exploring the current situation of herbaria and their use, and the situation of Botany as a field in Argentina, we raised a series of questions that served as a guide to search for information, and to construct databases and their subsequent analysis.

How is the population of herbaria-users characterized in terms of demographic indicators such as age, gender, geographic distribution, affiliations, among others? What use is herbarium data to researchers? What are the most frequent and infrequent herbarium-based research areas? Which are the preferred journals chosen by local botanists? What is the impact of these publications? Regarding the use of herbaria, which are the more consulted journals? And what is the status of the digitalization process in local herbaria? How are they linked among researchers and publications, and with researchers abroad? These questions seek, on the one hand, to characterize botanists as part of the scientific community in Argentina, and also to explore the scientific publications that cite the herbaria, and establish collaborative networks both locally and internationally.

To address the guide questions, two local databases were explored: The Argentine Science and Technology Information Portal (SiCyTAR, Sistema de Información de Ciencia y Tecnología Argentino, http://datos.mincyt.gob.ar/#/) and the CONICET Search Engine (https://www.conicet.gov.ar/new_scp/advancedsearch.php). The Argentine Science and Technology Information Portal gathers information on who, what, where, when, why, and how science, technology and innovation are done, and updates statistical data. The focus is on active researchers—researchers being defined in a broad sense as those who have published at least 5 academic papers during the analyzed period—affiliated to an Argentine institution, and that mention in their public CV at least one of the following keywords: botanist, Botany, herbaria, herbarium, herbarium collections, plant science, systematics, taxonomist, taxonomy, type specimen (both in English and Spanish). Filters were applied to stretch the search and to exclude other sciences such as Microbiology or Zoology. Afterwards, the questions were presented, and an answer was given using the information provided by the analyses of the databases and some references obtained in the bibliography.

### The Population of Herbaria-Users

In order to characterize the population of herbaria-users characterized in terms of demographic indicators, we explored the SiCyTAR database, and found that 5.5% of the results obtained were biologists, of which 1.1% corresponded to botanists, following the global tendency (Woodland, 2007). Sixty-six per cent of the botanists were women and 52% were under 50 years old. Forty-one percent of them worked in the Province of Buenos Aires (including the city of Buenos Aires). Of the total number of researchers, 27% were affiliated to a University. To narrow down the search, focus was put on researchers affiliated to CONICET. In the last 15 years, the number of CONICET researchers has increased from 4,000 to 10,000. Biologists constituted 10.6% of the total (including all subdisciplines) and, of this population, only 4.3% were botanists (0.46% of all the researchers, or 51 people).

Nowadays, the proportion of women in CONICET overpasses the proportion of men. When considering the whole of the researchers of this institution, and distributing them according to the hierarchical categories in which they are located, the percentages of women are the following: assistant researcher: 60.65% (out of 3169 researchers); associate researcher: 55.01% (out of 3463); independent researcher: 49.02% (out of 2370); principal researcher: 41.60% (out of 1048), and superior researcher: 25.36% (out of 205) (https://cifras.conicet.gov.ar/publica/detalle-tags/3). Only the last three categories enable the researchers to be part of the selection and evaluation committees and to become an Institute Director as other decision-making positions in academia. This gender bias is also a global tendency (Larivière et al., 2013) since it reflects the same pattern reported for academics in the field of Natural Sciences in particular, where women are under-represented in high ranks of scientific institutions, and also in editorial boards (Cho et al., 2014; European Commission, 2016).

Gender bias is part of a Western ‘cultural cognitive model’ that has distorted science (Howard-Borjas, 2001). A global report from UNESCO (2007) has highlighted that women are less likely to become scientist than men. Nowadays, female researchers publish less and with lower impact than their male colleagues (Larivière et al., 2013; Cislak et al., 2018). Furthermore, there are examples that women are under-paid compared to men (Shen, 2013), and that their research is less likely to receive funding (Ley and Hamilton, 2008; Ranga et al., 2012). Although Botany has historically been seen as suitable for women (Lindon et al., 2015), our results show a strong gender bias, which is also a global tendency among natural science and academia. Other studies within Botany’s subdisciplines have arrived at similar conclusions, e.g. nomenclature (Lindon et al., 2015) and ethnobotany not only considering women as authors but also as a subject of study (Howard-Borjas, 2001; Howard, 2006).

### The Uses of Herbaria Data and Herbarium-Based Research Areas

The most frequent results for herbarium-based research areas, extracted from SiCyTAR database are: Agriculture, Agricultural Biotechnology, Animal Production, Biological Sciences, Chemical Sciences, Computer and Information Sciences, Education Sciences, Forestry and Fisheries, Health Sciences, Earth Sciences and Veterinary. However, other fields are also listed: Art, Chemical Engineering, Civil Engineering, Economics and Business, Ethics and Religion, History and Archeology, Language and Literature, Law, Mathematics, Philosophy and Sociology. Examples of these less conventional areas are the study of the pre-Columbian arts, the use of materials for the construction of houses, the settlement of the Jesuits in Argentina (and Latin America) and the study of the natural and cultural heritage of native communities.

### Most Preferred Journals of Publication and Their Impact

To explore global tendencies, we chose Scopus. Scopus is the largest abstract and citation database of peer-reviewed literature (https://www.scopus.com). Information from Scopus was extracted from articles, reviews, articles in press, book chapters, letters and notes with the following combination of keywords (in English) that could be present in the title, abstract or keywords: either botany or plant science, plus herbaria, herbarium, collections, nomenclature, taxonomy and/or type specimen. The search was restricted to documents containing at least one author affiliated to an Argentine Institution. Data in Scopus was available for the period between 1993 and 2018, resulting in 1917 documents from 530 sources.

**Figure 1:**
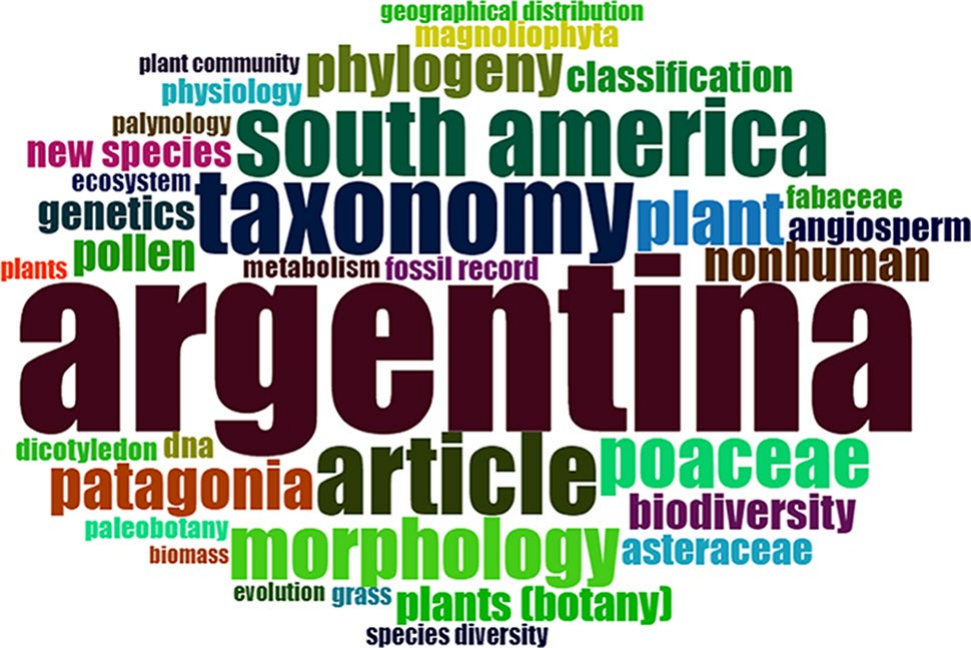
Keywords cloud based on the bibliometric analysis of publications stored at Scopus database from 1993 to 2018 (refer to the text to see filters of the search). Size of the word indicates the frequency of the word in the abstract and/or title.

**Figure 2:**
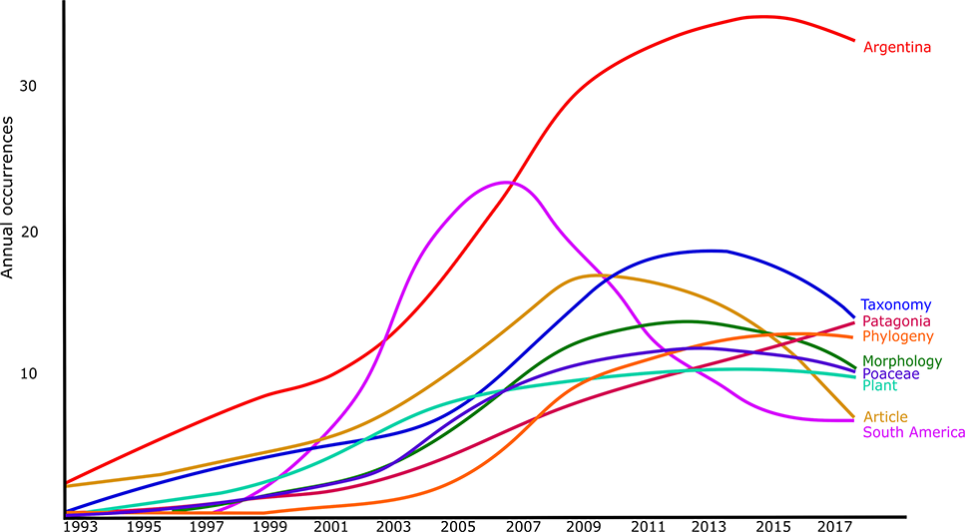
The tendency along time of the occurrence of the nine most frequent words in the abstract and/or title of herbaria-based-research articles, based on the bibliometric analysis of publications stored at Scopus database from 1993 to 2018 (refer to the text to see filters of the search).

The database was firstly visually inspected and filtered searching for outliers. It was then analyzed using the “Bibliometrix” package and the Biblioshiny app (Aria and Cuccurullo, 2017), implemented in the R environment (R CoreTeam, 2016). As expected, the use of herbaria was principally linked with Systematic Botany, particularly with Taxonomy. This was easily reflected when analyzing the most relevant keywords, where phylogeny, taxonomy and morphology were the most frequent ones (excluding the words used as filters of our study: Argentina, South America, and plant, **Figure 1**). Moreover, Poaceae was one of the most frequent keywords, together with Patagonia. When analyzing the preferred keywords through time, the most frequent ones nowadays presented a negative slope except for Patagonia **Figure 2**. This can reflect the world tendency to spread the traditional uses of Systematics to include or collaborate with other fields (e.g. Ecology, Genetics, etc.).

**Figure 3:**
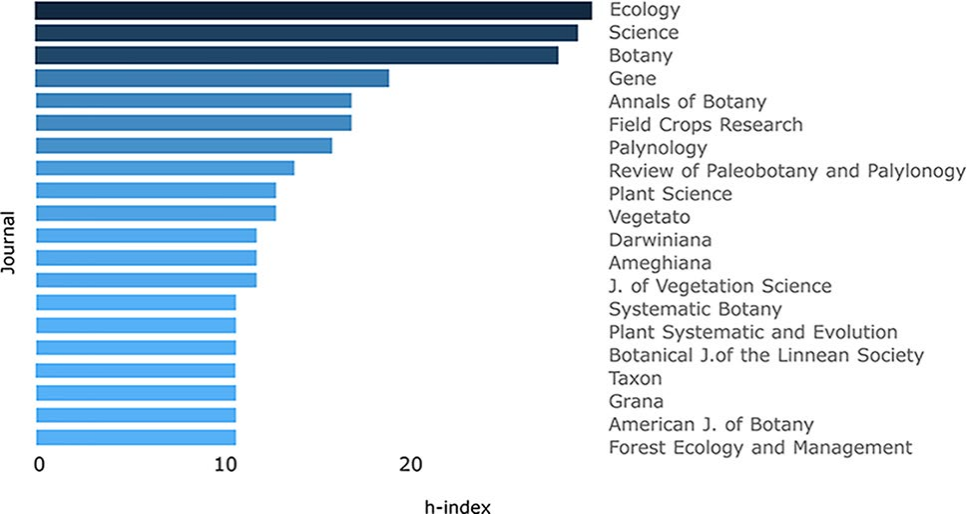
Journal’s impact (h-index) of the first 20 journals chosen to publish herbaria-based-research, based on the bibliometric analysis of publications stored at Scopus database from 1993 to 2018 (refer to the text to see filters of the search).

**Figure 4:**
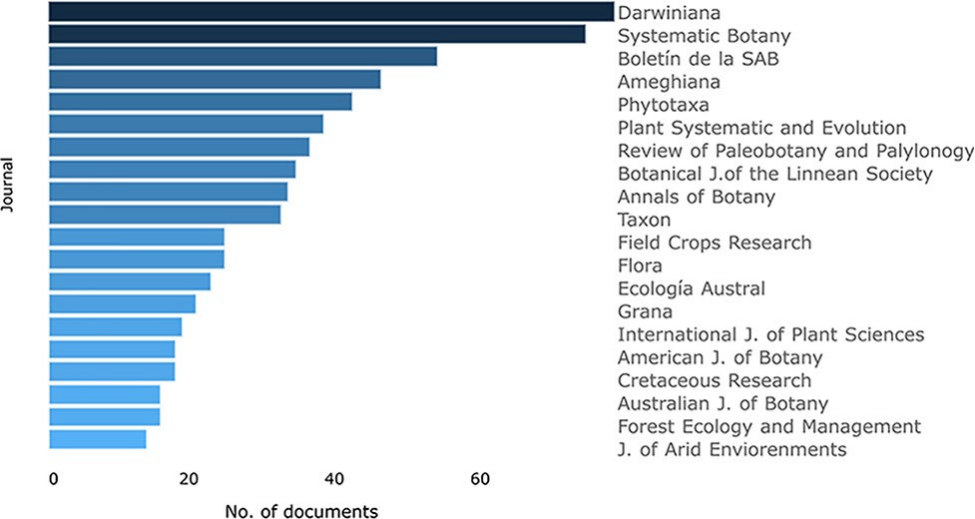
The number of herbaria-based-research articles published from 1993 to 2018 in the first 20 journals, based on the bibliometric analysis of publications stored at Scopus database from 1993 to 2018 (refer to the text to see filters of the search).

To analyze the impact of the publications, the H-index [system of measurement of quality based on the number of citations that a scientific article receives (Hirsch, 2005)] was considered. Ecology, Science and Botany were the sources that showed the highest impact and the lowest frequency in our database, in contrast with more frequent journals that showed a lower H-index (**Figure 3**). The preferred journal resulted in Darwiniana (Darwiniana+Darwiniana Nueva Serie), the second one was Systematic Botany and the third one, the “Boletín de la Sociedad Argentina de Botánica” (**Figure 4**). Darwiniana and the “Boletín” accept papers both in Spanish and English, likely one of the reasons for the researchers for choosing these journals, not only because of the facility of publishing in the native language but also because of the scope. For instance, if a floristic work is published it would be useful not only for the academic community, but also for agronomists, gardeners, and local public institutions that may be interested in these publications, and therefore more likely published in Spanish. Moreover, economic reasons are also listed as part of the election of these local journals (http://www2.darwin.edu.ar/). Among the most relevant foreign journals, Systematic Botany (position 2), Phytotaxa (position 4), Plant Systematics and Evolution (position 5), and Review of Paleobotany and Palynology (position 6) have been found. Taxon (position 10) and American Journal of Botany (position 18) were previously recovered as core journals chosen to publish articles of taxonomic botany (Walton and Morris, 2013).

**Figure 5:**
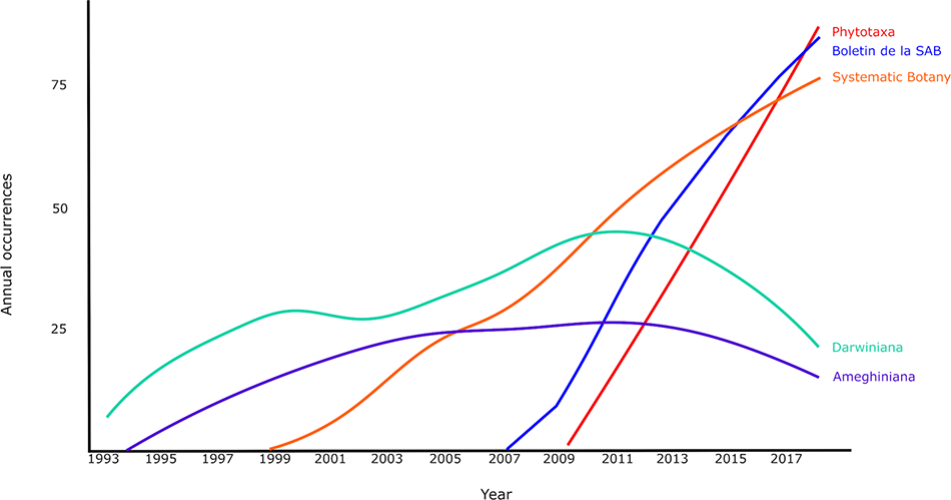
Dynamics along time of the more frequent journal chosen to publish herbaria-based-research published from 1993 to 2018, based on the bibliometric analysis of publications stored at Scopus database (refer to the text to see filters of the search).

When analyzing the tendency over time, the cumulative source growth reflects the same pattern observed for the most frequently used journals. However, when analyzing the source growth per year, the “Boletín de la Sociedad Argentina de Botánica” grows continuously and Systematic Botany also grows but with a lower slope (**Figure 5**). Since 2010, Darwiniana starts to have a negative slope while Phytotaxa grows steadily since its beginning (2011). Argentine researchers are changing their preferred journals for publication in the field of Botany as well as in other fields. The evaluation system tends to weigh the publications considering not only the number of published papers and their contribution but also the impact factor of the journal (Reyes et al., 2018).

### The Most Consulted Herbaria

With the information that Darwiniana is the preferred journal, the focus was thus on the use of national and international herbaria by analyzing the articles published in this journal. Darwiniana Journal originated in 1922, associated with the Darwinion Institute of Botany. Through the decades, the journal gained prestige and by 2004 a bibliometric study indicated that 90% of the articles published between 1998 and 2003 were sent by authors that were affiliated to other institutions (http://www2.darwin.edu.ar/). In 2013, Darwiniana, Kurtziana, Hickenia and Lorentziana, four botanical journals of recognized trajectory, were merged into Darwiniana “Nueva Serie” (New Series). After analyzing the articles published from 2013 to 2018—all the available data online and also the period after the fusion—the current uses of herbaria were recovered. Most of the articles were carried on by at least one Argentine author, but there were also authors associated with institutions of Brazil, Bolivia, Perú, and Chile. From the total number of articles published, 80% included material studied from at least one herbarium. Articles mentioned from one to 31 different herbaria with a median of 5, of which 36% were Argentine. When focusing on Argentine institutions, the citations ranged from 0 to 12. The most frequent ones resulted to be SI (17%), LIL and LP (12% each), and CORD and CTES (10%). The most cited herbaria are in concordance with the largest ones and those contributing to JSTOR with digital images of specimens (https://plants.jstor.org/). The number of cited herbaria has significantly increased from the inclusion of worldwide plant collections in the well-known JSTOR where botanists can access with a payed-membership and explore digital images of the specimens. To date, 9 Argentine herbaria contribute to this database (**Table 3**).

**Table 3:** Digitalization status of Argentine Vascular Plant collections.

Herbaria acronym	Digitalized specimens	Total specimens	% of digitalization
BA^+^	1500	242000	0.62
BAA	4738	200000	2.37
BAB	726	480000	0.15
BAF	304	400000	0.08
BAL	16	13200	0.12
CORD	12160	450000	2.70
CTES	2548	600000	0.42
LIL	4000	720000	0.56
LP	5171	400000	1.29
SI	28976.00	700000	4.14
Total	60139	4192000	1.43

^+^not contributing to JSTOR.

### The Impact of Digitalization in Local Herbaria

Herbarium collections encompassed the primarily source of plant distribution information through time (Nualart et al., 2017; Canteiro et al., 2019). The digitalization of the collections is taking the use of herbarium specimens to the next level, together with data aggregators (such as GBIF), herbaria are open to a wider public, and during the latter years novel herbaria based research is growing as well as research applied to conservation is improving (Lavoie, 2013; Greve et al., 2016; Soltis, 2017; Nualart et al., 2017). When accurately identified, each herbarium specimen is the registry of the presence of taxa at a specific location and time (Canteiro et al., 2019). The herbaria are not only a record for the existence of taxa but also enable the comparison among different periods of time and habitats, such as the study of flowering periods [e.g. (Guerreiro, 2014)], areas of endemism (Godoy-Bürki et al., 2014; Godoy Bürki et al., 2018), etc.

When searching digitalized images of specimens from Argentina within JSTOR we found that the herbarium specimens are stored at 111 herbaria; only 9 Argentine herbaria are Partners of the Global Plant Initiative contributing with ca. 60000 specimens (**Table 3**). When the results were analyzed for each herbarium, we noted that the digitalization process is still in its first steps, since the herbaria presenting the highest percentage of images correspond only to 4%.

### Scientific Networks and Co-Autorships

**Figure 6:**
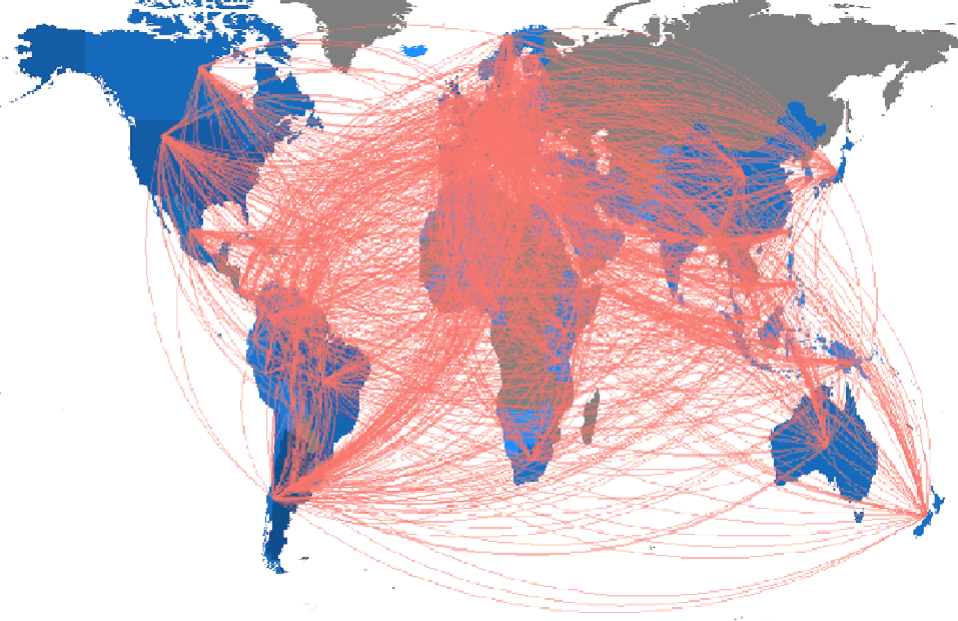
Collaboration map between Argentina and the rest of the world, based on herbaria-based-research articles published from 1993 to 2018, based on the bibliometric analysis of publications stored at Scopus (refer to the text to see filters of the search).

Half of the corresponding authors (ca. 1,600) of the analyzed documents are researchers affiliated with an Argentine institution, sharing co-authorships with researchers from the United States of America, Brazil, and Spain. When analyzing collaborations at the country level, researchers affiliated to Argentine institutions interact principally with those from the USA, Brazil, Spain, Germany, Chile, and Mexico, but also with researchers from countries of Africa, Asia and Oceania. There are no collaborations recovered from Russia (**Figure 6**). Regarding collaboration networks, the average number of authors per document is 4, less than the estimated in other bibliometric studies (e.g. Sauermann and Haeussler, 2017). It is important to highlight that in the analyzed period there are also changes in global tendencies. In the last decades, Botany has become highly collaborative and multidiscipline (Cellinese and Beaman, 2012), reflected by the gradual increase in the mean number of authors per article (Gu, 2004).

## Herbaria Uses Within Systematic Botany and Taxonomy

Over time, the herbaria were maintained as scientific institutions and social spaces. With the advances of new technologies, access to digitized collections, and the availability of a big amount of data, herbaria-based research is changing. However, the exchange of material and researches remains necessary. Next in this article, three cases of herbaria use within Systematic Botany and Taxonomy are explored.

### The Inventory and Categorization of Biodiversity

As a global tendency, several articles have pinpointed the problems faced by natural history collections, including herbaria, as well as the relegation of taxonomy as a discipline (Dubois, 2003; Funk, 2004; Kholia and Fraser-Jenkins, 2011). Herbaria are the institutions where the development of young taxonomists occurs, and the inventory of the plant diversity is kept. Hence the vital importance of herbaria in the development of plant inventories (e.g. floras, catalogs), the construction of taxonomic revisions and, consequently, the categorization of taxa, as well as in the development of conservation strategies (Schatz, 2002).

The knowledge of the native biodiversity of South America is necessary to carry out sustainable activities, as well as to preserve biodiversity. The Shenzhen Declaration on Plant Sciences states the necessity of contributing to the inventory of life on Earth in order to (i) know what is urgent to preserve, and (ii) learn about the unknowns before they become extinct (Shenzhen Declaration Drafting Committee, 2017). The botanists of the world, who have endorsed the Declaration, still believe that time, although short, does exist for answers to be found and solutions to be implemented (Kress and Knapp, 2017). Beyond academic purposes, floristic inventories are also helpful to local activities (e.g. in the identification of seeds, native and exotic plants, crops) and contribute to the increment of plant knowledge. Furthermore, they reinforce the collaboration among botanical institutes, and strengthen the institutional activities (e.g. facilitate the digitization processes of collections, illustrations, databases).

In South America, a big effort has been made to organize the Catalogues (e.g. Catálogo de Plantas Vasculares del Cono Sur, Catálogo de Plantas Vasculares de Bolivia), but most of the Flora systematization is still under construction [e.g. Brazil, Flora 2020 (Luiza et al., 2018; “http://floradobrasil.jbrj.gov.br” ), Flora of the Venezuelan Guayana (Steyermark et al., 1995)]. After several attempts, the “Flora Argentina” project is currently being published. The work is divided into 20 volumes, of which half have been already published (http://www.floraargentina.edu.ar).

For several reasons, the preparation of a list of threatened plant species has been more problematic than for other biological groups (Delucchi, 2006). Nowadays, Argentina lacks a list of plant conservation status, while for other taxonomic groups it has already been completed (e.g. Ojeda et al., 2000). In 2018, the Environment and Sustainable Development Office (in Spanish: Secretaría de Ambiente y Desarrollo Sustentable) has started efforts to get together national and international taxonomic specialists (principally associated to herbaria) to begin categorization based on UICN criteria (UICN-Comisión de Supervivencia de Especies de la UICN, 2001) [e.g. South Africa categorization, (von Staden et al., 2013)].

### Conservation of Biodiversity in Herbaria

Argentina has a tradition of grazing livestock, being the fifth largest producer of cattle in the world. The Pampas region produces 61% of the total beef cattle in the country, with more than 80% allocated to internal consumption (Rótolo et al., 2007). Potential for expanding exports has created incentives for increasing production and the consequent result on the expansion of the agricultural frontiers. In particular, the increasing global demand for soy products is the major driver for the extensive deforestation in the Neotropical dry forest ecosystems, including Argentina (Grau et al., 2008). Argentina’s Pampas region accounts for more than 90% of national grain production, with soybean, wheat, maize and sunflower as the main crops (Magrin et al., 2005). The Pampas region counts with few phylogeographic studies on native flora (see **Figure 1** Fregonezi et al., 2013). Several authors have pinpointed that the Pampas have only been preserved in areas not suitable for agriculture due to unfavorable edaphic or climatic conditions (Baldi et al., 2006), such as the Pampas Hills (Tandilia and Ventania systems) or flooding lands. Furthermore, the region encompasses few conserved areas (e.g. “Parque Provincial Ernesto Tornquist”) and, in general, includes introduced plants and cattle (e.g. Cuevas and Zalba, 2009; Cuevas, 2010).

Here is where the conservation of biodiversity in herbaria (as well as the preservation in the Genbank, Botanical Gardens, etc.) is crucial. For decades, the herbarium collections have served as a valuable first step to identify species of interest for conservation and to highlight information gaps that required additional research. Herbaria have been useful tools to identify rare plant taxa, to group taxa by habitat affinity, to refine their conservation status based on the vulnerability of the habitats (MacDougall et al., 1998; Magrini and Scoppola, 2010), and also to predict the species distribution before field trips (Guisan and Thuiller, 2005).

### The Study of Crop Wild Relatives

Crop Wild Relatives (CWR) are species closely related to crops, including crop progenitors, and are defined by their potential ability to contribute with beneficial traits to crops such as pest or disease resistance, yield improvement or stability. Crop wild relatives contribute to food security and sustainable environments. The global conservation strategies include ex situ (conservation outside their natural habitats) and *in situ* (conservation in their natural surroundings) maintenance. Like any other group of wild species, CWR are subject to an increasing range of threats in their host habitats, and thus more systematic attention to their conservation is required (Maxted and Kell, 2009).

In Argentina, one of these CWR are the wild potato species that have been included as a priority in the global strategy for conservation of crop wild relatives, and therefore both *in situ* (primarily in protected areas) and ex situ conservation programs were implemented (Clausen et al., 2018). For the *ex situ* conservation, in the INTA Active Germplasm Bank (https://inta.gob.ar/documentos/banco-activo-de-germoplasma-de-la-eea-balcarce), the material collected as the botanical seed is regenerated and preserved as such and forms a Collection of Seeds of Wild Potato Species. On the other hand, the material collected in a vegetative state constitutes the *in vitro* collection of potato conformed by Andean potato varieties, clones of the INTA Potato Genetic Improvement Program, specific genotypes of wild potato species and work collections. The BAL Herbarium had its origin as the place to store the specimens that were collected along with field trips for the Germplasm Bank. However, the role of the Herbarium as a part of an *ex situ* conservation strategy has not been fully exploited yet. For this reason, it is necessary to emphasize that the collection can be used to (1) make distribution maps of the CWR; (2) plan prospecting and collection campaigns; (3) characterize the accompanying flora; (4) perform predictive modeling of distribution and macro-ecological niche modeling that can support ecological and evolutionary studies; (5) serve as backup in the case of finding inconsistencies in the conserved material once the regeneration is carried out and (6) serve as support for molecular and morphological characterizations.

## Concluding Remarks and Future Perspectives

Herbaria collections play a key role in botanical research. In Argentina, the discipline is weakened since botanists represent only 5% of the population of biologists, following the global tendency. Reviewing the history of the discipline, analyzing the botanists as a community and the herbaria as a social space, enables us to think about the uses of herbaria in research and other fields. Herbaria-based research is being reconfigured and herbaria as institutions adapt to changes. Internationalization of publications and network collaboration between people and disciplines are the strongest current trends. Many large herbaria have already implemented digitalization goals; meanwhile, the small local herbaria should also work in that way. Although there are limited resources for the maintenance of natural collections, it is important to rescue the key role of herbaria-based research in the inventory of the local flora, and in the design of conservation strategies to preserve biodiversity in times of climate change. Efforts should be focused on training new generations in the appreciation of plants, and in generating scientific based resources available for the decision makers in public policies. We, as a community, must be open to new perspectives and accept other worldviews to enrich our practices as botanists and herbaria users.

## Author Contributions

AL conceived the idea, and AS improved it. AL and AS constructed the databases. AS performed the statistical analysis and the graphics. AL and AS analyzed the data and co-wrote the manuscript.

## Funding

AL acknowledges the National Research Council of Argentina (CONICET) as researcher, and AS as post-doctoral fellowship holder.

## Conflict of Interest

The authors declare that the research was conducted in the absence of any commercial or financial relationships that could be construed as a potential conflict of interest.
